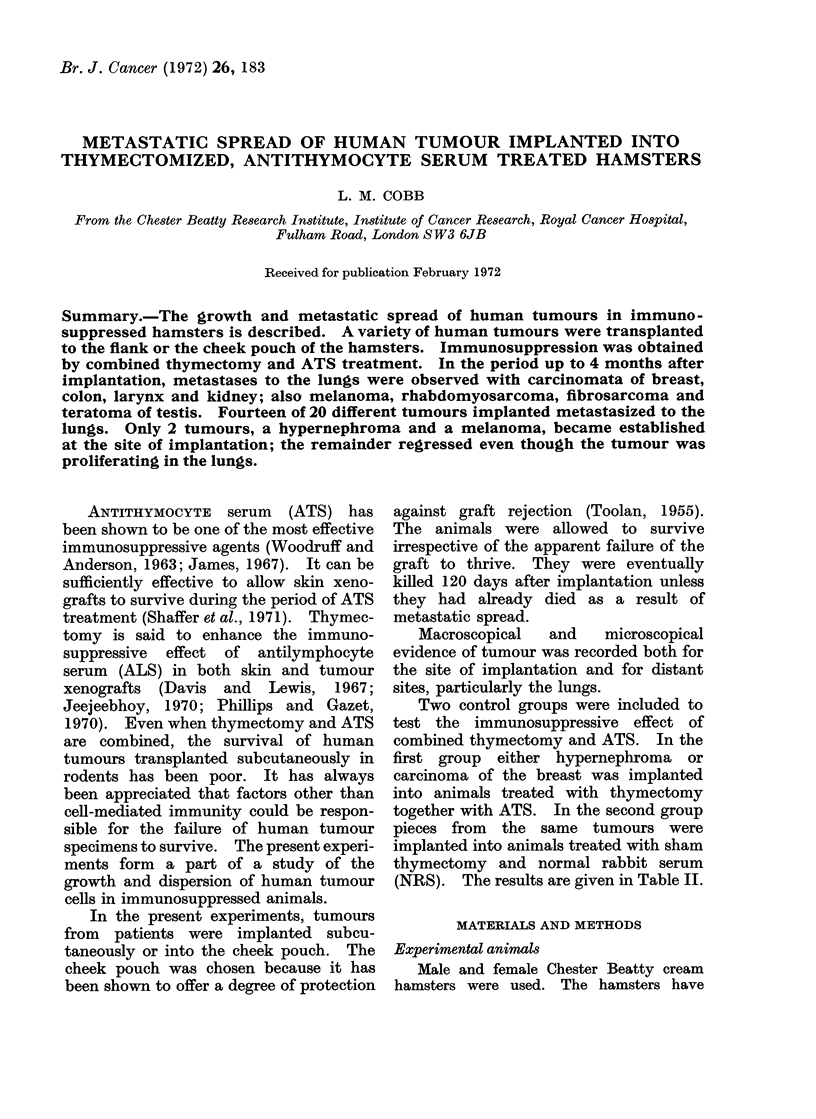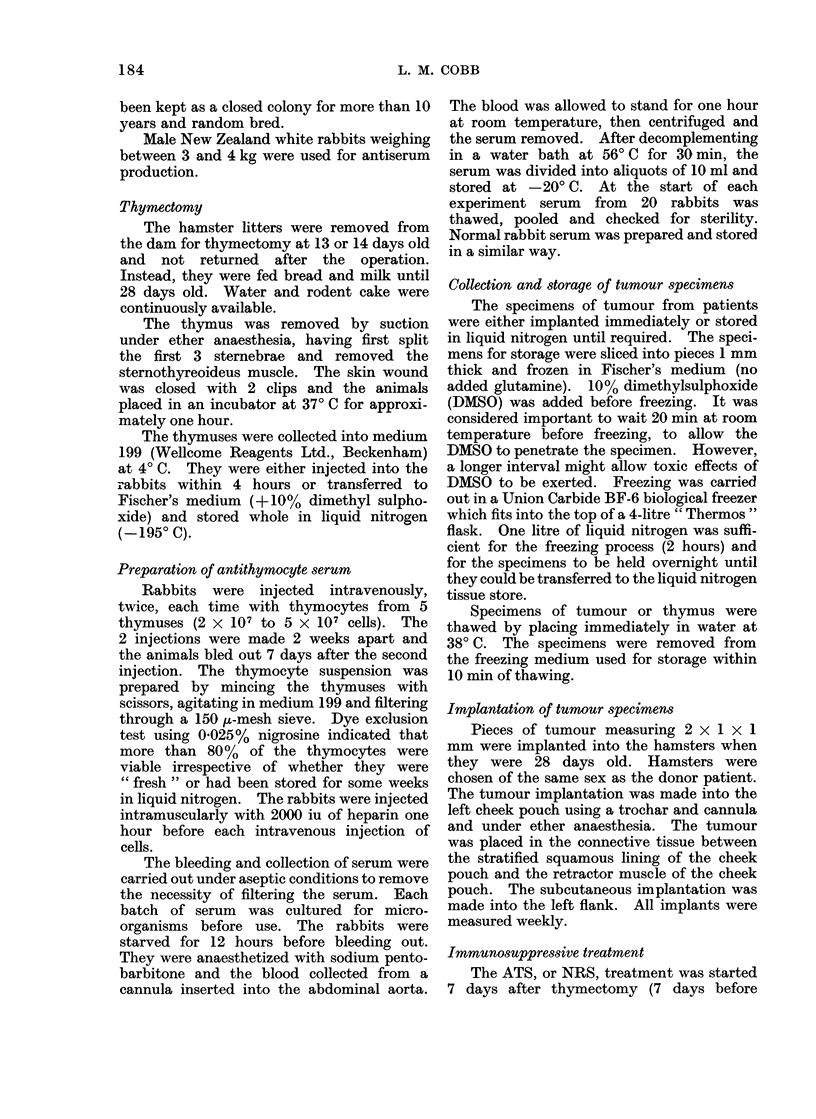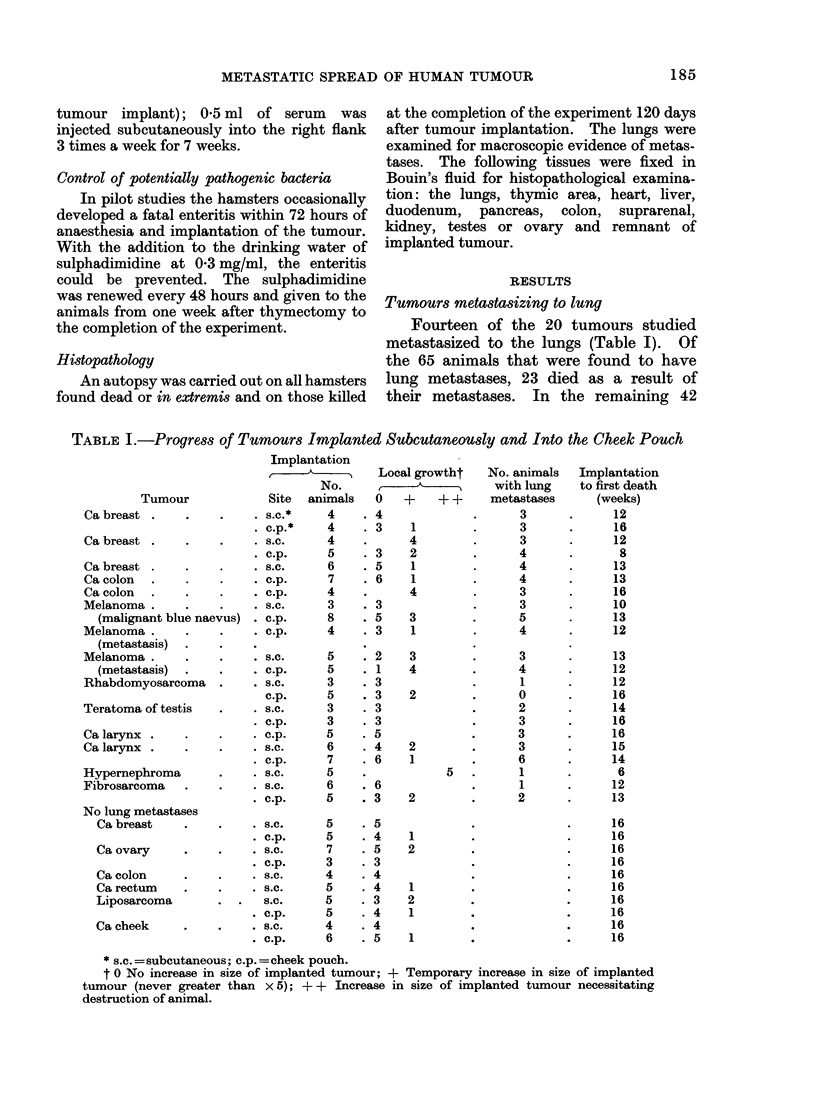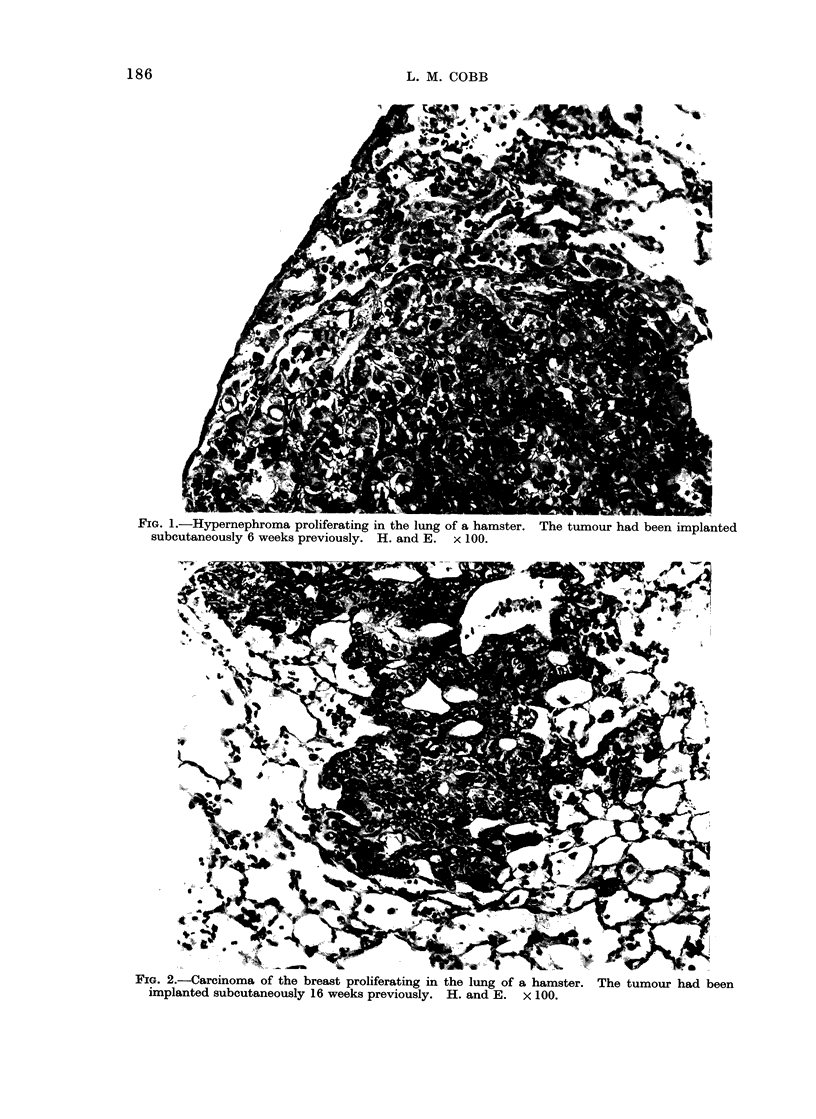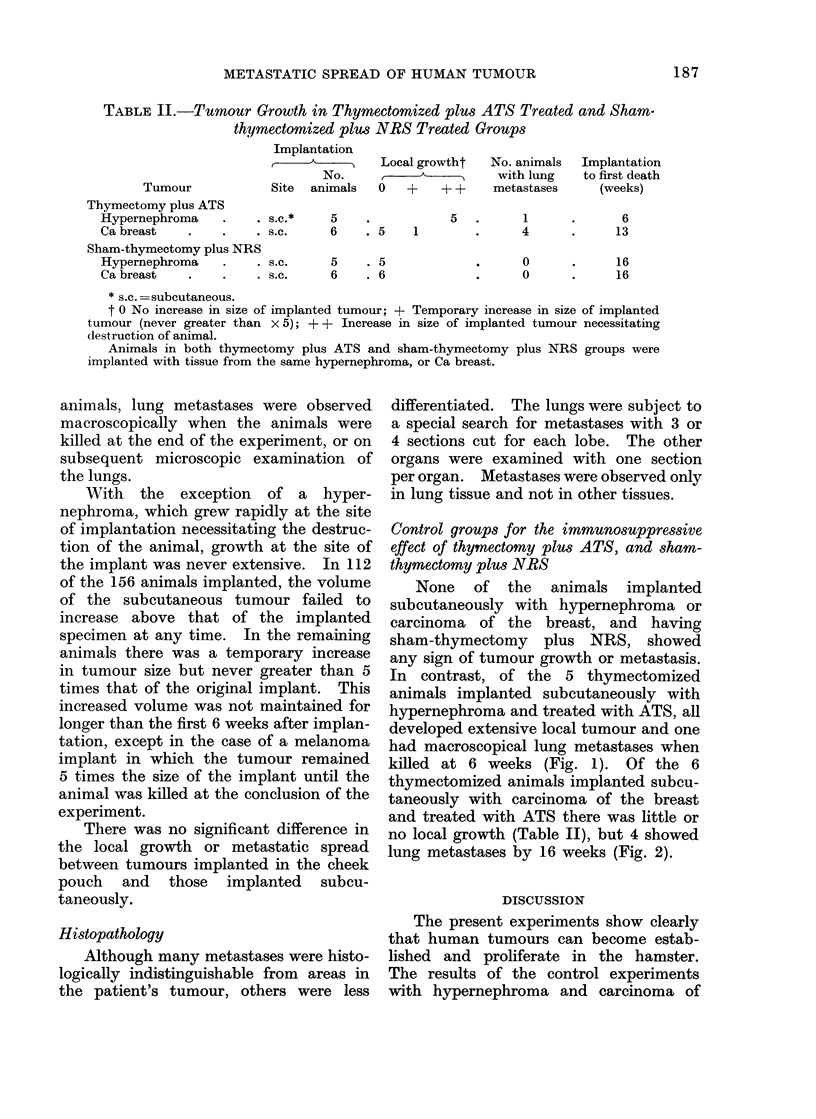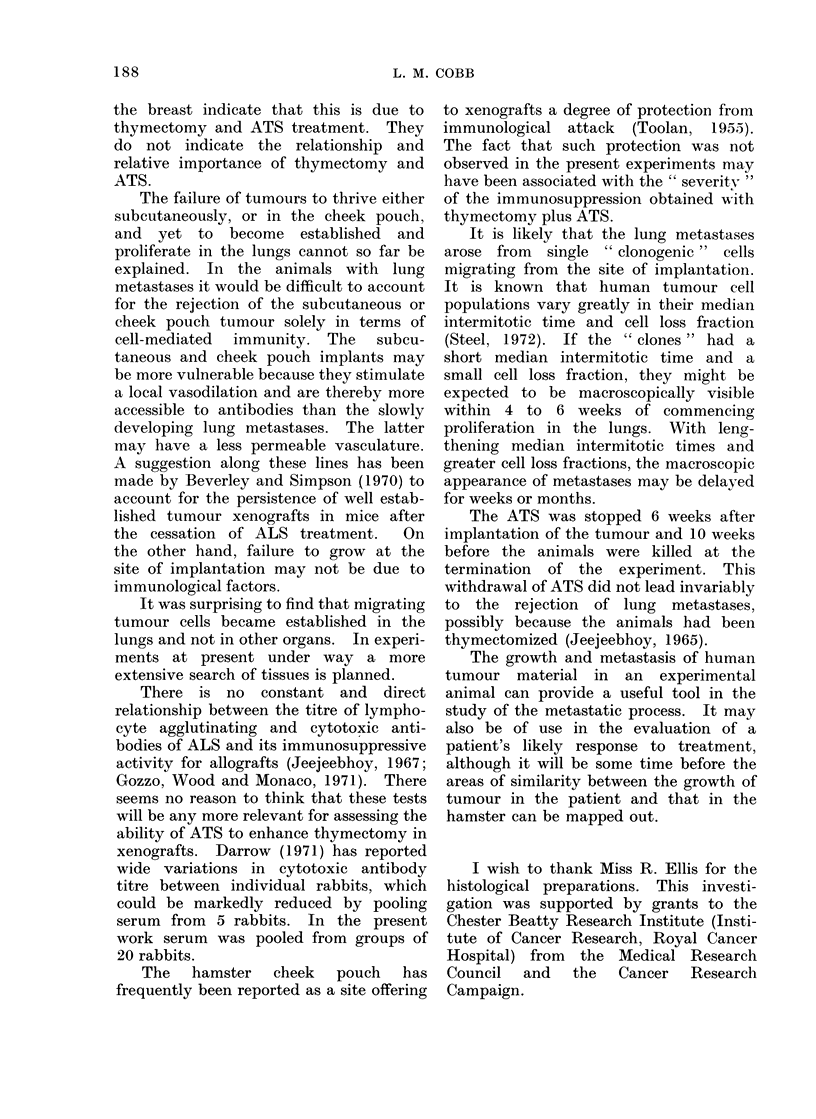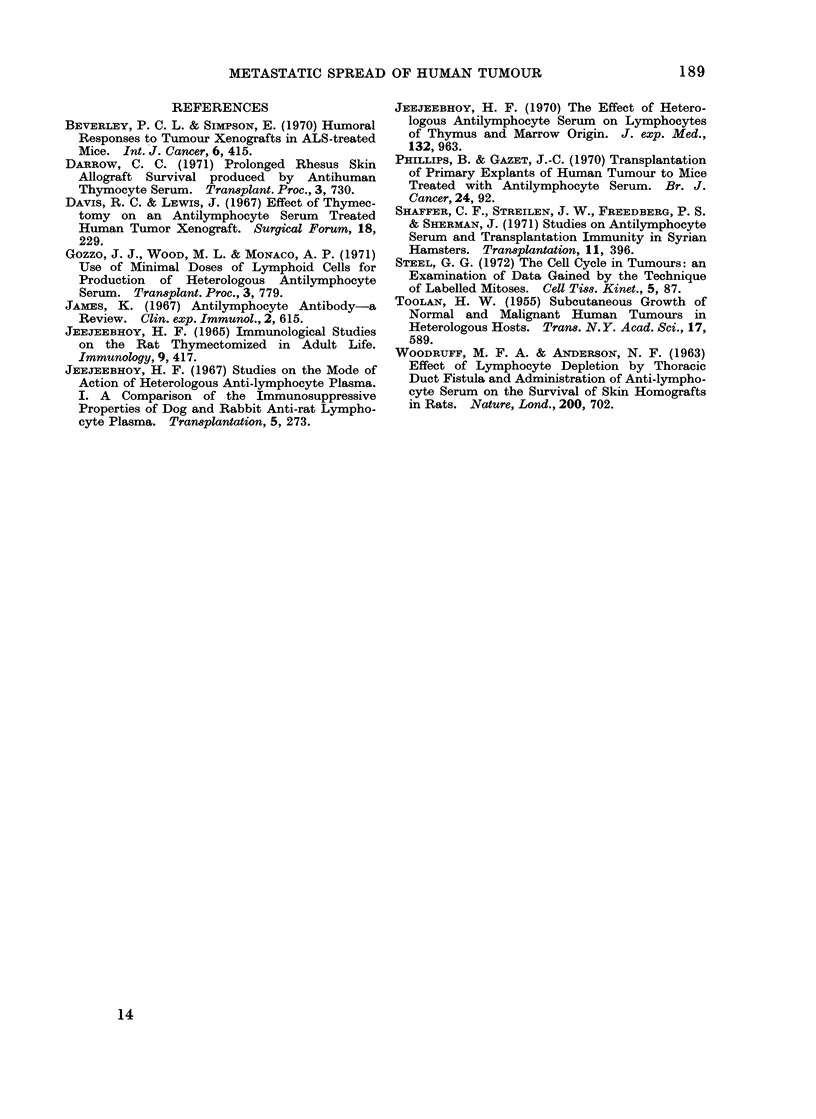# Metastatic Spread of Human Tumour Implanted into Thymectomized, Antithymocyte Serum Treated Hamsters

**DOI:** 10.1038/bjc.1972.25

**Published:** 1972-06

**Authors:** L. M. Cobb

## Abstract

**Images:**


*Br. J. Cancer* (1972) **26**, 183

### METASTATIC SPREAD OF HUMAN TUMOUR IMPLANTED INTO THYMECTOMIZED, ANTITHYMOCYTE SERUM TREATED HAMSTERS

L. M. COBB

*From the Chester Beatty Research Institute, Institute of Cancer Research, Royal Cancer Hospital, Fulham Road, London SW3 6JB*

Received for publication February 1972

**Summary.—The growth and metastatic spread of human tumours in immunosuppressed hamsters is described. A variety of human tumours were transplanted to the flank or the cheek pouch of the hamsters. Immunosuppression was obtained by combined thymectomy and ATS treatment. In the period up to 4 months after implantation, metastases to the lungs were observed with carcinomata of breast, colon, larynx and kidney; also melanoma, rhabdomyosarcoma, fibrosarcoma and teratoma of testis. Fourteen of 20 different tumours implanted metastasized to the lungs. Only 2 tumours, a hypernephroma and a melanoma, became established at the site of implantation; the remainder regressed even though the tumour was proliferating in the lungs.**

ANTITHYMOCYTE serum (ATS) has been shown to be one of the most effective immunosuppressive agents (Woodruff and Anderson, 1963; James, 1967). It can be sufficiently effective to allow skin xenografts to survive during the period of ATS treatment (Shaffer *et al.*, 1971). Thymectomy is said to enhance the immunosuppressive effect of antilymphocyte serum (ALS) in both skin and tumour xenografts (Davis and Lewis, 1967; Jeejeebhoy, 1970; Phillips and Gazet, 1970). Even when thymectomy and ATS are combined, the survival of human tumours transplanted subcutaneously in rodents has been poor. It has always been appreciated that factors other than cell-mediated immunity could be responsible for the failure of human tumour specimens to survive. The present experiments form a part of a study of the growth and dispersion of human tumour cells in immunosuppressed animals.

In the present experiments, tumours from patients were implanted subcutaneously or into the cheek pouch. The cheek pouch was chosen because it has been shown to offer a degree of protection against graft rejection (Toolan, 1955). The animals were allowed to survive irrespective of the apparent failure of the graft to thrive. They were eventually killed 120 days after implantation unless they had already died as a result of metastatic spread.

Macroscopical and microscopical evidence of tumour was recorded both for the site of implantation and for distant sites, particularly the lungs.

Two control groups were included to test the immunosuppressive effect of combined thymectomy and ATS. In the first group either hypernephroma or carcinoma of the breast was implanted into animals treated with thymectomy together with ATS. In the second group pieces from the same tumours were implanted into animals treated with sham thymectomy and normal rabbit serum (NRS). The results are given in Table II.

### MATERIALS AND METHODS

### *Experimental animals*

Male and female Chester Beatty cream hamsters were used. The hamsters have been kept as a closed colony for more than 10 years and random bred.

Male New Zealand white rabbits weighing between 3 and 4 kg were used for antiserum production.

*Thymectomy*

The hamster litters were removed from the dam for thymectomy at 13 or 14 days old and not returned after the operation. Instead, they were fed bread and milk until 28 days old. Water and rodent cake were continuously available.

The thymus was removed by suction under ether anaesthesia, having first split the first 3 sternebrae and removed the sternothyreoideus muscle. The skin wound was closed with 2 clips and the animals placed in an incubator at 37° C for approximately one hour.

The thymuses were collected into medium 199 (Wellcome Reagents Ltd., Beckenham) at 4° C. They were either injected into the rabbits within 4 hours or transferred to Fischer's medium (+10% dimethyl sulphoxide) and stored whole in liquid nitrogen (−195° C).

*Preparation of antithymocyte serum*

Rabbits were injected intravenously, twice, each time with thymocytes from 5 thymuses ($2 \times 10^7$ to $5 \times 10^7$ cells). The 2 injections were made 2 weeks apart and the animals bled out 7 days after the second injection. The thymocyte suspension was prepared by mincing the thymuses with scissors, agitating in medium 199 and filtering through a 150 $\mu$-mesh sieve. Dye exclusion test using 0·025% nigrosine indicated that more than 80% of the thymocytes were viable irrespective of whether they were " fresh " or had been stored for some weeks in liquid nitrogen. The rabbits were injected intramuscularly with 2000 iu of heparin one hour before each intravenous injection of cells.

The bleeding and collection of serum were carried out under aseptic conditions to remove the necessity of filtering the serum. Each batch of serum was cultured for microorganisms before use. The rabbits were starved for 12 hours before bleeding out. They were anaesthetized with sodium pentobarbitone and the blood collected from a cannula inserted into the abdominal aorta. The blood was allowed to stand for one hour at room temperature, then centrifuged and the serum removed. After decomplementing in a water bath at 56° C for 30 min, the serum was divided into aliquots of 10 ml and stored at −20° C. At the start of each experiment serum from 20 rabbits was thawed, pooled and checked for sterility. Normal rabbit serum was prepared and stored in a similar way.

*Collection and storage of tumour specimens*

The specimens of tumour from patients were either implanted immediately or stored in liquid nitrogen until required. The specimens for storage were sliced into pieces 1 mm thick and frozen in Fischer's medium (no added glutamine). 10% dimethylsulphoxide (DMSO) was added before freezing. It was considered important to wait 20 min at room temperature before freezing, to allow the DMSO to penetrate the specimen. However, a longer interval might allow toxic effects of DMSO to be exerted. Freezing was carried out in a Union Carbide BF-6 biological freezer which fits into the top of a 4-litre " Thermos " flask. One litre of liquid nitrogen was sufficient for the freezing process (2 hours) and for the specimens to be held overnight until they could be transferred to the liquid nitrogen tissue store.

Specimens of tumour or thymus were thawed by placing immediately in water at 38° C. The specimens were removed from the freezing medium used for storage within 10 min of thawing.

*Implantation of tumour specimens*

Pieces of tumour measuring $2 \times 1 \times 1$ mm were implanted into the hamsters when they were 28 days old. Hamsters were chosen of the same sex as the donor patient. The tumour implantation was made into the left cheek pouch using a trochar and cannula and under ether anaesthesia. The tumour was placed in the connective tissue between the stratified squamous lining of the cheek pouch and the retractor muscle of the cheek pouch. The subcutaneous implantation was made into the left flank. All implants were measured weekly.

*Immunosuppressive treatment*

The ATS, or NRS, treatment was started 7 days after thymectomy (7 days before tumour implant); 0·5 ml of serum was injected subcutaneously into the right flank 3 times a week for 7 weeks.

*Control of potentially pathogenic bacteria*

In pilot studies the hamsters occasionally developed a fatal enteritis within 72 hours of anaesthesia and implantation of the tumour. With the addition to the drinking water of sulphadimidine at 0·3 mg/ml, the enteritis could be prevented. The sulphadimidine was renewed every 48 hours and given to the animals from one week after thymectomy to the completion of the experiment.

*Histopathology*

An autopsy was carried out on all hamsters found dead or *in extremis* and on those killed at the completion of the experiment 120 days after tumour implantation. The lungs were examined for macroscopic evidence of metastases. The following tissues were fixed in Bouin's fluid for histopathological examination: the lungs, thymic area, heart, liver, duodenum, pancreas, colon, suprarenal, kidney, testes or ovary and remnant of implanted tumour.

### RESULTS

*Tumours metastasizing to lung*

Fourteen of the 20 tumours studied metastasized to the lungs (Table I). Of the 65 animals that were found to have lung metastases, 23 died as a result of their metastases. In the remaining 42

TABLE I.—*Progress of Tumours Implanted Subcutaneously and Into the Cheek Pouch*

| | Implantation | | Local growth† | | | | |
|---|---|---|---|---|---|---|---|
| Tumour | Site | No. animals | 0 | + | ++ | No. animals with lung metastases | Implantation to first death (weeks) |
| Ca breast | s.c.* | 4 | 4 | | | 3 | 12 |
| | c.p.* | 4 | 3 | 1 | | 3 | 16 |
| Ca breast | s.c. | 4 | | 4 | | 3 | 12 |
| | c.p. | 5 | 3 | 2 | | 4 | 8 |
| Ca breast | s.c. | 6 | 5 | 1 | | 4 | 13 |
| Ca colon | c.p. | 7 | 6 | 1 | | 4 | 13 |
| Ca colon | c.p. | 4 | | 4 | | 3 | 16 |
| Melanoma | s.c. | 3 | 3 | | | 3 | 10 |
| (malignant blue naevus) | c.p. | 8 | 5 | 3 | | 5 | 13 |
| Melanoma | c.p. | 4 | 3 | 1 | | 4 | 12 |
| (metastasis) | | | | | | | |
| Melanoma | s.c. | 5 | 2 | 3 | | 3 | 13 |
| (metastasis) | c.p. | 5 | 1 | 4 | | 4 | 12 |
| Rhabdomyosarcoma | s.c. | 3 | 3 | | | 1 | 12 |
| | c.p. | 5 | 3 | 2 | | 0 | 16 |
| Teratoma of testis | s.c. | 3 | 3 | | | 2 | 14 |
| | c.p. | 3 | 3 | | | 3 | 16 |
| Ca larynx | c.p. | 5 | 5 | | | 3 | 16 |
| Ca larynx | s.c. | 6 | 4 | 2 | | 3 | 15 |
| | c.p. | 7 | 6 | 1 | | 6 | 14 |
| Hypernephroma | s.c. | 5 | | | 5 | 1 | 6 |
| Fibrosarcoma | s.c. | 6 | 6 | | | 1 | 12 |
| | c.p. | 5 | 3 | 2 | | 2 | 13 |
| No lung metastases | | | | | | | |
| Ca breast | s.c. | 5 | 5 | | | | 16 |
| | c.p. | 5 | 4 | 1 | | | 16 |
| Ca ovary | s.c. | 7 | 5 | 2 | | | 16 |
| | c.p. | 3 | 3 | | | | 16 |
| Ca colon | s.c. | 4 | 4 | | | | 16 |
| Ca rectum | s.c. | 5 | 4 | 1 | | | 16 |
| Liposarcoma | s.c. | 5 | 3 | 2 | | | 16 |
| | c.p. | 5 | 4 | 1 | | | 16 |
| Ca cheek | s.c. | 4 | 4 | | | | 16 |
| | c.p. | 6 | 5 | 1 | | | 16 |

* s.c. = subcutaneous; c.p. = cheek pouch.

† 0 No increase in size of implanted tumour; + Temporary increase in size of implanted tumour (never greater than ×5); ++ Increase in size of implanted tumour necessitating destruction of animal.

FIG. 1.—Hypernephroma proliferating in the lung of a hamster. The tumour had been implanted subcutaneously 6 weeks previously. H. and E. ×100.

FIG. 2.—Carcinoma of the breast proliferating in the lung of a hamster. The tumour had been implanted subcutaneously 16 weeks previously. H. and E. ×100.

TABLE II.—*Tumour Growth in Thymectomized plus ATS Treated and Sham-thymectomized plus NRS Treated Groups*

| Tumour | Implantation Site | Implantation No. animals | Local growth† 0 | Local growth† + | Local growth† ++ | No. animals with lung metastases | Implantation to first death (weeks) |
|---|---|---|---|---|---|---|---|
| Thymectomy plus ATS | | | | | | | |
| Hypernephroma | s.c.* | 5 | | | 5 | 1 | 6 |
| Ca breast | s.c. | 6 | 5 | 1 | | 4 | 13 |
| Sham-thymectomy plus NRS | | | | | | | |
| Hypernephroma | s.c. | 5 | 5 | | | 0 | 16 |
| Ca breast | s.c. | 6 | 6 | | | 0 | 16 |

* s.c. = subcutaneous.

† 0 No increase in size of implanted tumour; + Temporary increase in size of implanted tumour (never greater than × 5); ++ Increase in size of implanted tumour necessitating destruction of animal.

Animals in both thymectomy plus ATS and sham-thymectomy plus NRS groups were implanted with tissue from the same hypernephroma, or Ca breast.

animals, lung metastases were observed macroscopically when the animals were killed at the end of the experiment, or on subsequent microscopic examination of the lungs.

With the exception of a hypernephroma, which grew rapidly at the site of implantation necessitating the destruction of the animal, growth at the site of the implant was never extensive. In 112 of the 156 animals implanted, the volume of the subcutaneous tumour failed to increase above that of the implanted specimen at any time. In the remaining animals there was a temporary increase in tumour size but never greater than 5 times that of the original implant. This increased volume was not maintained for longer than the first 6 weeks after implantation, except in the case of a melanoma implant in which the tumour remained 5 times the size of the implant until the animal was killed at the conclusion of the experiment.

There was no significant difference in the local growth or metastatic spread between tumours implanted in the cheek pouch and those implanted subcutaneously.

*Histopathology*

Although many metastases were histologically indistinguishable from areas in the patient's tumour, others were less differentiated. The lungs were subject to a special search for metastases with 3 or 4 sections cut for each lobe. The other organs were examined with one section per organ. Metastases were observed only in lung tissue and not in other tissues.

*Control groups for the immunosuppressive effect of thymectomy plus ATS, and sham-thymectomy plus NRS*

None of the animals implanted subcutaneously with hypernephroma or carcinoma of the breast, and having sham-thymectomy plus NRS, showed any sign of tumour growth or metastasis. In contrast, of the 5 thymectomized animals implanted subcutaneously with hypernephroma and treated with ATS, all developed extensive local tumour and one had macroscopical lung metastases when killed at 6 weeks (Fig. 1). Of the 6 thymectomized animals implanted subcutaneously with carcinoma of the breast and treated with ATS there was little or no local growth (Table II), but 4 showed lung metastases by 16 weeks (Fig. 2).

### DISCUSSION

The present experiments show clearly that human tumours can become established and proliferate in the hamster. The results of the control experiments with hypernephroma and carcinoma of the breast indicate that this is due to thymectomy and ATS treatment. They do not indicate the relationship and relative importance of thymectomy and ATS.

The failure of tumours to thrive either subcutaneously, or in the cheek pouch, and yet to become established and proliferate in the lungs cannot so far be explained. In the animals with lung metastases it would be difficult to account for the rejection of the subcutaneous or cheek pouch tumour solely in terms of cell-mediated immunity. The subcutaneous and cheek pouch implants may be more vulnerable because they stimulate a local vasodilation and are thereby more accessible to antibodies than the slowly developing lung metastases. The latter may have a less permeable vasculature. A suggestion along these lines has been made by Beverley and Simpson (1970) to account for the persistence of well established tumour xenografts in mice after the cessation of ALS treatment. On the other hand, failure to grow at the site of implantation may not be due to immunological factors.

It was surprising to find that migrating tumour cells became established in the lungs and not in other organs. In experiments at present under way a more extensive search of tissues is planned.

There is no constant and direct relationship between the titre of lymphocyte agglutinating and cytotoxic antibodies of ALS and its immunosuppressive activity for allografts (Jeejeebhoy, 1967; Gozzo, Wood and Monaco, 1971). There seems no reason to think that these tests will be any more relevant for assessing the ability of ATS to enhance thymectomy in xenografts. Darrow (1971) has reported wide variations in cytotoxic antibody titre between individual rabbits, which could be markedly reduced by pooling serum from 5 rabbits. In the present work serum was pooled from groups of 20 rabbits.

The hamster cheek pouch has frequently been reported as a site offering to xenografts a degree of protection from immunological attack (Toolan, 1955). The fact that such protection was not observed in the present experiments may have been associated with the "severity" of the immunosuppression obtained with thymectomy plus ATS.

It is likely that the lung metastases arose from single "clonogenic" cells migrating from the site of implantation. It is known that human tumour cell populations vary greatly in their median intermitotic time and cell loss fraction (Steel, 1972). If the "clones" had a short median intermitotic time and a small cell loss fraction, they might be expected to be macroscopically visible within 4 to 6 weeks of commencing proliferation in the lungs. With lengthening median intermitotic times and greater cell loss fractions, the macroscopic appearance of metastases may be delayed for weeks or months.

The ATS was stopped 6 weeks after implantation of the tumour and 10 weeks before the animals were killed at the termination of the experiment. This withdrawal of ATS did not lead invariably to the rejection of lung metastases, possibly because the animals had been thymectomized (Jeejeebhoy, 1965).

The growth and metastasis of human tumour material in an experimental animal can provide a useful tool in the study of the metastatic process. It may also be of use in the evaluation of a patient's likely response to treatment, although it will be some time before the areas of similarity between the growth of tumour in the patient and that in the hamster can be mapped out.

I wish to thank Miss R. Ellis for the histological preparations. This investigation was supported by grants to the Chester Beatty Research Institute (Institute of Cancer Research, Royal Cancer Hospital) from the Medical Research Council and the Cancer Research Campaign.

### REFERENCES

Beverley, P. C. L. & Simpson, E. (1970) Humoral Responses to Tumour Xenografts in ALS-treated Mice. *Int. J. Cancer*, **6**, 415.

Darrow, C. C. (1971) Prolonged Rhesus Skin Allograft Survival produced by Antihuman Thymocyte Serum. *Transplant. Proc.*, **3**, 730.

Davis, R. C. & Lewis, J. (1967) Effect of Thymectomy on an Antilymphocyte Serum Treated Human Tumor Xenograft. *Surgical Forum*, **18**, 229.

Gozzo, J. J., Wood, M. L. & Monaco, A. P. (1971) Use of Minimal Doses of Lymphoid Cells for Production of Heterologous Antilymphocyte Serum. *Transplant. Proc.*, **3**, 779.

James, K. (1967) Antilymphocyte Antibody—a Review. *Clin. exp. Immunol.*, **2**, 615.

Jeejeebhoy, H. F. (1965) Immunological Studies on the Rat Thymectomized in Adult Life. *Immunology*, **9**, 417.

Jeejeebhoy, H. F. (1967) Studies on the Mode of Action of Heterologous Anti-lymphocyte Plasma. I. A Comparison of the Immunosuppressive Properties of Dog and Rabbit Anti-rat Lymphocyte Plasma. *Transplantation*, **5**, 273.

Jeejeebhoy, H. F. (1970) The Effect of Heterologous Antilymphocyte Serum on Lymphocytes of Thymus and Marrow Origin. *J. exp. Med.*, **132**, 963.

Phillips, B. & Gazet, J.-C. (1970) Transplantation of Primary Explants of Human Tumour to Mice Treated with Antilymphocyte Serum. *Br. J. Cancer*, **24**, 92.

Shaffer, C. F., Streilen, J. W., Freedberg, P. S. & Sherman, J. (1971) Studies on Antilymphocyte Serum and Transplantation Immunity in Syrian Hamsters. *Transplantation*, **11**, 396.

Steel, G. G. (1972) The Cell Cycle in Tumours: an Examination of Data Gained by the Technique of Labelled Mitoses. *Cell Tiss. Kinet.*, **5**, 87.

Toolan, H. W. (1955) Subcutaneous Growth of Normal and Malignant Human Tumours in Heterologous Hosts. *Trans. N.Y. Acad. Sci.*, **17**, 589.

Woodruff, M. F. A. & Anderson, N. F. (1963) Effect of Lymphocyte Depletion by Thoracic Duct Fistula and Administration of Anti-lymphocyte Serum on the Survival of Skin Homografts in Rats. *Nature, Lond.*, **200**, 702.